# Maximum static inspiratory and expiratory pressures with different lung volumes

**DOI:** 10.1186/1475-925X-5-29

**Published:** 2006-05-05

**Authors:** Christopher G Lausted, Arthur T Johnson, William H Scott, Monique M Johnson, Karen M Coyne, Derya C Coursey

**Affiliations:** 1 The Institute for Systems Biology, 1441 North 34^th ^Street, Seattle, WA 98103, USA; 2 Biological Resources Engineering, University of Maryland, College Park, MD 20742, USA; 3 GE Healthcare Technologies, 3114 N. Grandview Blvd W-553, Waukesha, WI 53188, USA; 4 U.S. Army Edgewood CB Center, APG, MD 21010, USA

## Abstract

**Background:**

Maximum pressures developed by the respiratory muscles can indicate the health of the respiratory system, help to determine maximum respiratory flow rates, and contribute to respiratory power development. Past measurements of maximum pressures have been found to be inadequate for inclusion in some exercise models involving respiration.

**Methods:**

Maximum inspiratory and expiratory airway pressures were measured over a range of lung volumes in 29 female and 19 male adults. A commercial bell spirometry system was programmed to occlude airflow at nine target lung volumes ranging from 10% to 90% of vital capacity.

**Results:**

In women, maximum expiratory pressure increased with volume from 39 to 61 cmH_2_O and maximum inspiratory pressure decreased with volume from 66 to 28 cmH_2_O. In men, maximum expiratory pressure increased with volume from 63 to 97 cmH_2_O and maximum inspiratory pressure decreased with volume from 97 to 39 cmH_2_O. Equations describing pressures for both sexes are:

P_e_/P_max _= 0.1426 Ln( %VC) + 0.3402 R^2 ^= 0.95

P_i_/P_max _= 0.234 Ln(100 - %VC) - 0.0828 R^2 = ^0.96

**Conclusion:**

These results were found to be consistent with values and trends obtained by other authors. Regression equations may be suitable for respiratory mechanics models.

## Background

While maximum respiratory pressures at the mouth have been measured in numerous subjects, less data exists to characterize maximum pressures as they vary with lung volume. Maximum pressure is volume dependent because muscle tension is length dependent, because muscle tension produces higher pressure with a smaller radius of curvature, and because respiratory tissue is elastic. Rahn et al. [1] first produced static pressure-volume diagrams from a group of adult men, and later, Cook et al. [2] produced pressure-volume diagrams from a larger group of subjects including women and children. These diagrams were useful in modeling the energetics of respiration [3] and in monitoring the progress of respiratory muscle training [4]. Yet the total number of subjects tested remained small, particularly regarding females. The present paper provides additional static pressure-volume data obtained from adult volunteers, both women and men.

## Methods

### Subjects

Forty-eight normal subjects agreed to participate in the study. The subjects were recruited from students and staff at the University of Maryland. The study was approved by the Institutional Review Board and all subjects gave informed consent. The subjects' characteristics are shown in Table 1.

**Table 1:** Age, height, weight, and lung volume data in the two groups tested shown with standard deviations.

Group	Number of Subjects	Average Age (Range)	Average Height, cm	Average Mass kg	Average Vital Capacity, L
Females	29	25.5 ± 3.7 (18 to 31)	164 ± 6	60.5 ± 13.3	3.58 ± 0.63
Males	19	26.5 ± 4.3 (19 to 34)	177 ± 7	74.1 ± 8.8	4.72 ± 8.4

### Protocol

Subjects were first acquainted with the spirometer and the test protocol. They were instructed in the definition of functional residual capacity (FRC) as the resting volume of the lung and given time to practice finding FRC. The subjects were then measured for inspiratory capacity (IC) and expiratory reserve volume (ERV) relative to FRC. Volume measurements were repeated until three consecutive maneuvers produced volumes within a 100 ml range. The average of the three volumes was recorded. Vital capacity (VC) was calculated as the sum of IC and ERV.

Maximum pressure measurements were taken from occlusions occurring at nine predetermined target volumes. The volumes were randomly ordered and ranged from 10% to 90% of VC by 10% increments. Subjects began each maneuver at FRC. Subjects were instructed to inhale or exhale, as necessary, to the desired volume. When the target volume was obtained, the occlusion valve automatically closed. The subject was told to inhale or exhale, as necessary, for two seconds. After two seconds of effort, the valve was released. This gave the subject access to fresh air for at least one minute of rest. More time was given, if desired. After all 18 measurements were taken, the test was repeated. The stronger effort, or higher maximum pressure, at each measurement was saved.

### Measurements

A commercial spirometery system (Collins™, Braintree, MA) was used for all the measurements. Lung volumes were monitored by the dry-seal bell spirometer. The occlusion valve and pressure transducer utilized were those located in what Collins refers to as its "universal breathing valve." Collins "Research Assistant" (RA) software controlled the occlusion valve and collected pressure and volume measurements. For the experiment, a supervisory program, "PV", was authored in Microsoft Visual Basic for Applications^™ ^to configure RA and provide feedback to the experimenters. PV was designed to fill the spirometer with an appropriate volume of fresh air prior to each measurement. It then calculated the spirometer volume corresponding to the target lung volume, taking thermal expansion into account. Occlusion was triggered automatically when the subject reached the target lung volume. As target lung volumes were randomly ordered, a randomization feature was built into PV. After each occlusion, RA returned pressure and volume data, which PV analyzed and saved to disk. The spirometer was calibrated each test day with a three-liter syringe and the pressure transducer was calibrated each day with a 10 cmH_2_O manometer.

### Data analysis

The maximum inhalation or exhalation pressure magnitude (P_i _or P_e_) at each lung volume (V_L_) was recorded by the computer. The average pressure for the last one second of each effort was calculated. This pressure was then used to correct V_L _using the method by Cook et al. [2]. Absolute lung volumes were not measured and volumes were calculated based on the assumption that residual volume was 26% that of TLC.

## Results

Average maximum pressure values for all of the female subjects tested appear in Table 2 and values for all of the male subjects appear in Table 3. Observations were grouped according to the lung volumes at which occlusion occurred and actual volumes within each group were averaged to produce the tabled values. The pressures produced by the men were typically 64% higher than the women in expiration, and 53% higher in inspiration.

**Table 2:** Maximal inspiratory and expiratory static pressures at different lung volumes for the female subjects.

Expiratory	Pressure		Inspiratory	Pressure	
Volume (%VC)	Positive Pressure (cmH_2_O)	P_e_/P_max_	Volume (%VC)	Negative Pressure (cmH_2_O)	P_i_/P_max_

8.9 ± 0.7	38.7 ± 25.0	0.5864	12.1 ± 0.9	65.9 ± 31.6	0.9985
18.4 ± 1.0	44.2 ± 25.9	0.6697	22.5 ± 1.3	65.0 ± 31.6	0.9848
27.6 ± 1.3	53.4 ± 30.7	0.8091	33.0 ± 1.8	59.6 ± 32.0	0.9030
37.3 ± 1.5	53.7 ± 28.8	0.8136	43.3 ± 2.0	55.0 ± 32.0	0.8333
46.8 ± 1.9	53.3 ± 28.9	0.8076	53.5 ± 2.2	53.0 ± 32.9	0.8030
56.2 ± 2.2	55.4 ± 29.6	0.8394	63.8 ± 2.6	47.6 ± 28.3	0.7212
65.7 ± 2.6	57.3 ± 33.2	0.8682	73.6 ± 2.6	42.3 ± 28.6	0.6409
74.7 ± 3.2	61.7 ± 38.5	0.9398	83.1 ± 2.7	34.2 ± 27.4	0.5182
84.4 ± 3.2	61.2 ± 39.0	0.9273	92.6 ± 2.4	28.0 ± 29.0	0.4242

Volumes are expressed in percent of vital capacity at ambient pressure. All values are shown with standard deviations. P_max _for females was determined to be 66 cmH_2_O.

**Table 3:** Maximal inspiratory and expiratory static pressure at different lung volumes for the male subjects.

Expiratory	Pressure		Inspiratory	Pressure	
Volume (%VC)	Positive Pressure (cmH_2_O)	P_e_/P_max_	Volume (%VC)	Negative Pressure (cmH_2_O)	P_i_/P_max_

8.2 ± 1.0	63.2 ± 32.6	0.6196	13.5 ± 2.0	96.7 ± 45.5	0.9480
17.2 ± 1.3	75.3 ± 39.0	0.7382	24.2 ± 2.8	95.3 ± 49.8	0.9343
26.2 ± 1.6	84.1 ± 39.9	0.8245	35.2 ± 3.2	97.3 ± 52.2	0.9539
35.2 ± 2.2	91.6 ± 38.3	0.8980	45.9 ± 3.6	87.2 ± 42.3	0.8549
44.8 ± 2.6	89.6 ± 41.3	0.8784	55.7 ± 3.2	77.9 ± 36.9	0.7637
53.5 ± 2.7	93.3 ± 36.3	0.9147	66.7 ± 4.2	75.4 ± 42.2	0.7392
62.6 ± 3.4	94.6 ± 44.3	0.9275	76.5 ± 4.5	69.4 ± 40.4	0.6804
72.1 ± 3.8	94.2 ± 43.3	0.9235	85.5 ± 4.9	51.4 ± 38.4	0.5039
80.8 ± 4.2	97.2 ± 41.5	0.9529	94.2 ± 5.1	38.9 ± 38.8	0.3814

Volumes are expressed in percent of vital capacity at ambient pressure. All values are shown with standard deviations. P_max _for males was determined to be 102 cmH_2_O.

Data were then scrutinized in an exploratory manner to see if they could be easily and universally fit by a simple mathematical expression [5]. It was found that both men's and women's data could be described by an expression of the form:

P_e_/P_max _= A Ln (%VC) + B for exhalation

and

P_i_/P_max _= C Ln (100 - %VC) + D for inhalation

Here, P_max _is the asymptotically maximum pressure that could be developed by the respiratory muscles at any lung volume and P_i _is the maximum inspiratory pressure that can be developed at specific lung volumes. The average value of P_max _found by determining the limit of the nonlinear P-V curve for the group of subjects was found to be 102 cmH_2_O for males and 66 cmH_2_O for females, and was found to be the same for both inhalation and exhalation directions. Least squares regression using Microsoft Excel yielded the following two equations:

P_e_/P_max _= 0.1426 Ln (%VC) + 0.3402 R^2 ^= 0.9549

and

P_i_/P_max _= 0.234 Ln (100% - %VC) - 0.0828 R^2 ^= 0.9642

**Figure 1:**
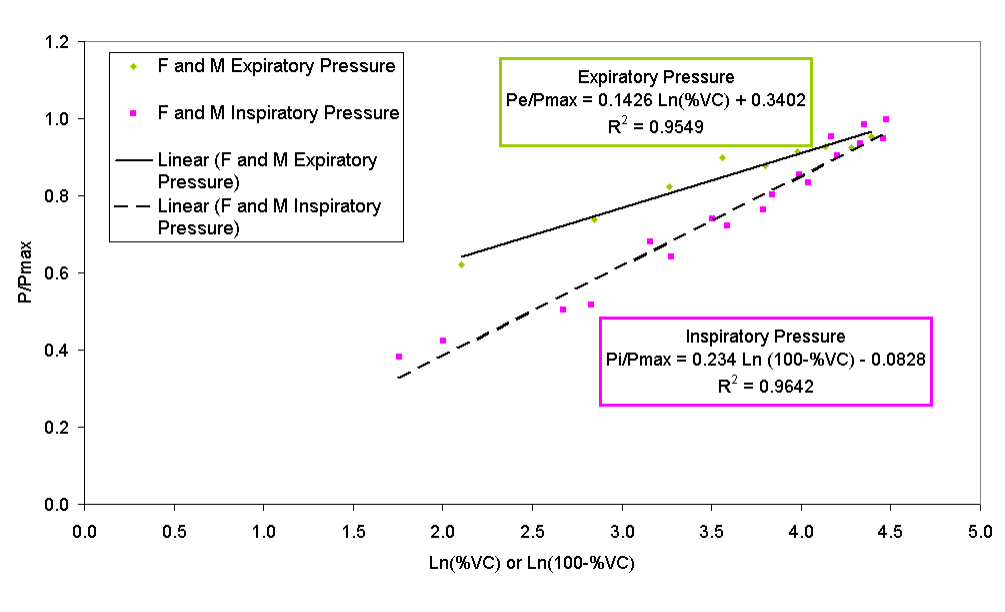
Graph of empirical equations determined to describe relative expiratory and inspiratory pressures for men and women. Some data points are coincident. Symbols: F = female; M = male; other symbols as in text.

These equations are graphed in Figure 1.

## Discussion

Greater (more positive) expiratory pressures were developed at higher V_L_, while greater (more negative) inspiratory pressures were developed at lower V_L_. In women P_e _increased with volume from 39 to 61 cmH_2_O and P_i _decreased with volume from 66 to 28 cmH_2_O. In men, P_e _increased with volume from 63 to 97 cmH_2_O and P_1 _decreased with volume from 97 to 39 cmH_2_O. These trends occur primarily for two reasons. First, respiratory muscles work both with and against respiratory tissue elastance to produce pressure. Expiratory efforts are aided by tissue elastance (lung recoil effects) at high V_L _and inhibited at low V_L_. Inspiratory efforts are inhibited by tissue elastance at high V_L _and aided at low V_L_. Second, respiratory muscles exert greater tension when they are stretched to greater lengths. Expiratory muscles are stretched when the lung is inflated, while inspiratory muscles are stretched when the lung is deflated. Both of these factors describe the general trend of the data.

The volume dependence of P_i _was much more pronounced than the volume dependence of P_e _in both women and men. This can be seen as a higher slope of the inspiratory equation compared to the expiratory equation in Figure 1. This may reflect a combination of strength differences between diaphragm (largely responsible for inhalation) and abdominal muscles (largely responsible for exhalation) recruitment of intercoastal muscle (largely responsible for posture maintenance), and different mechanical advantages of each type of muscle as the lung volume varies.

The Laplace equation may be relevant here. This equation states that enclosed pressure is proportional to the product of wall tension and wall thickness and inversely proportional to the radius of curvature. The Laplace equation for a sphere differs from that of a cylinder by a factor of two. Pressure in a sphere (P = ) is twice that of a cylinder (P = ), all other things being equal.

The diaphragm is positioned under the lungs and curves upward in a somewhat spherical shape. As it contracts, it becomes flatter, meaning that its radius of curvature increases. Lung volume increases as the diaphragm contracts. If the Laplace equation can be applied to the respiratory system, then it would show that inspiratory pressure should decrease as radius, and thus lung volume, increases (P_i _∝ , as long as wall tension and thickness remain steady).

The abdominal muscles are arranged differently, more like wrapping around a cylinder. The abdominal muscles flatten at smaller lung volumes instead of larger lung volumes, and the Laplace equation indicates that higher pressures should be developed at larger lung volumes (P ∝ V).

Both effects have been observed. Inspiratory pressures increase as lung volume decreases and expiratory pressure increases as lung volume increases. There is roughly a factor of two between the dependence of pressures upon lung volumes for inspiration and expiration. This could well be related to the difference in the Laplace equation for a sphere and a cylinder.

The pressure-volume data obtained in this study are of the same general magnitudes as those previously reported [1,2,6]. Rahn et al. [1] studied P_i _in 11 men and P_e _in 12 men using similar methods. The highest pressures from three efforts at each of six starting volumes were recorded. Measurements were read from a mercury manometer connected to the subjects' noses. Pressure-volume data closely match the results of the present study. Craig [6] produced pressure-volume diagrams from 10 men using methods similar to the present study. Pressures were taken from a mercury manometer connected to the subjects' mouths. These data also closely match the results of the present study. Cook et al. [2] studied 17 males and 9 females using two techniques. One technique was a conventional occlusion maneuver. The other technique involved subjects breathing into or out of large, fixed volumes. The compressibility of the air in differently sized containers provided for various ultimate lung volumes. The volumes were calculated from Boyle's Law using peak pressures that could be sustained for 1–2 seconds. Five volumes were used. It was concluded that the results of the occlusion method and the compression method were the same. In women, the compression-method P_i _values were similar to those of the present study at high volumes, but slightly higher at lower volumes. The P_e _values were similar at low volumes, but much higher at higher lung volumes. In men, the compression-method P_i _values agree well with those of the present study. However, the P_e _values are much higher than those of the present study at the higher volumes. Cook et al. [2] suggested that their P_e _values might have been higher than the Rahn et al. values because of the use of mouth pressure measurements rather than nose pressure measurements. It was also hypothesized that these P_e _values exceeded Craig's values due to better mouthpiece sealing.

As the results of this study are more in agreement with work of Rahn et al. [1] and Craig [6], it is more likely that there is another reason for the discrepancy. Aside from muscle strength alone, P_e _and P_i _are highly effort dependent. Subjects may limit their maximum pressures due to factors such as pain in the ear or general discomfort. During some maximum pressure maneuvers, researchers have observed changes in hemodynamics leading to loss of consciousness [7]. It is possible that the subjects of the Cook et al. [2] study were more highly motivated. It is also possible that these subjects were of above average strength.

Numerous authors have collected maximal pressures at a single V_L_. Most recently, Wilson et al. [8] measured maximal P_e _and P_i _in 87 women and 48 men using partial occlusion and Bourdon gauges. The women were found to have P_e _= 93 ± 17 cmH_2_O and P_i _= 73 ± 22 cmH_2_O and the men were found to have P_e _= 148 ± 34 cmH_2_O and P_i _= 106 ± 31 cmH_2_O. It could be expected that the P_i _and P_e _values from a single volume study would exceed the values from a multiple volume study because more efforts are made at the optimal V_L _in the single volume study, while muscle fatigue can be a factor in the multiple volume study.

Judging from inspiration values, this does not appear to be the case. In the present study, women were found to have P_i _= 66 ± 32 cmH_2_O at V_L _= 12%VC and men were found to have P_i _= 97 ± 46 cmH_2_O at V_L _= 14%VC. These values are virtually identical to the Wilson et al. [8] data. On the other hand, women in this study were found to have P_e _= 61 ± 39 cmH_2_O at V_L _= 84%VC and men were found to have P_e _97 ± 42 cmH_2_O at V_L _= 81%VC. These values are considerably smaller than the Wilson et al. data. The P_e _values of the single volume study fall in between the maximum P_e _values of the present study and the maximum P_e _values of the Cook et al. [2] study.

Satisfactorily describing maximum lung pressures with mathematical expressions can be helpful for respiratory mechanical modeling [3]. It is not likely that maximal pressures would be developed in young, healthy adults during quiet breathing. During exercise, and especially during expiratory flow limitation, however, maximum pressures may well be developed. For example, modeling the effects of respiratory masks during hard work could use these equations to calculate respiratory work rate. These equation forms are good because pressures and lung volumes both appear as relative rather than absolute values. That way, both men's and women's pressures could be determined with the same equations despite large differences in absolute pressures developed. Respiratory models for those conditions could well use the equations developed here.

Although we have no data to support the notion, it is possible, if their respiratory mechanics changed proportionally, that maximum pressures developed by patients with respiratory impairments could be described by the same equations as developed here. That is because these equations are in relative pressure and volume form. One would expect that P_max _could be much lower in diseased patients, but P/P_max _could be scaled the same. If this were so, then equations developed here could have more universal value.

## Conclusion

Maximum pressures at the mouth have been determined to depend on lung volumes. Equations to describe these pressures have been developed, and these are in a form that may be useful for modeling and predictive purposes.

## Abbreviations

ERV expiratory reserve volume of the lung, L

FRC functional residual capacity of the lung, L

IC inspiratory capacity of the lung, L

P_e _volume-dependent maximum expiratory pressure, cmH_2_O

P_i _volume-dependent maximum inspiratory pressure, cmH_2_O

P_max _volume-independent maximum pressure, cmH_2_O

PV name of supervisory computer program

RA proprietary data acquisition and analysis program from Collins

TLC total lung capacity, L

V_L _lung volume, LVC

VC vital capacity of the lung, L

## Competing interests

The author(s) declare that they have no competing interests.

## Authors' contributions

CGL wrote software and procedures, and conducted many of the tests. ATJ suggested the experiment, wrote much of the paper, and determined the form of equations. WHS provided support, including planning, protocol, and supplies. MMJ assisted with planning and conducted many of the tests. KMC assisted with planning and conducted many of the tests. DCC provided data analysis support.
